# A Comparison of Intra-Articular Blood Cell Secretome and Blood Cell Secretome with Triamcinolone Acetonide in Dogs with Osteoarthritis: A Crossover Study

**DOI:** 10.3390/ani12233358

**Published:** 2022-11-30

**Authors:** J. C. Alves, Ana Santos, Patrícia Jorge, L. Miguel Carreira

**Affiliations:** 1 Divisão de Medicina Veterinária, Guarda Nacional Republicana (GNR), Rua Presidente Arriaga, 9, 1200-771 Lisbon, Portugal; 2 MED—Mediterranean Institute for Agriculture, Environment and Development, Instituto de Investigação e Formação Avançada, Universidade de Évora, Pólo da Mitra, Ap. 94, 7006-554 Évora, Portugal; 3 Faculty of Veterinary Medicine, University of Lisbon (FMV/ULisboa), 1300-477 Lisbon, Portugal; 4 Interdisciplinary Centre for Research in Animal Health (CIISA), University of Lisbon (FMV/ULisboa), 1649-004 Lisboa, Portugal; 5 Anjos of Assis Veterinary Medicine Centre (CMVAA), 2830-077 Barreiro, Portugal

**Keywords:** Blood Cell Secretome, orthobiologic, triamcinolone, dog, osteoarthritis, pain, hip

## Abstract

**Simple Summary:**

Osteoarthritis is a very common joint disease in dogs. There are multiple treatments described for the disease, but there is still a need for treatments that change disease progression while managing symptoms. For that reason, a growing interest has arisen around biological treatments such as Blood Cell Secretome. The goal of this study was to compare the intra-articular administration of Blood Cell Secretome to its combined administration with triamcinolone in dogs with hip osteoarthritis. Our results show that Blood Cell Secretome improves the overall condition of OA patients and that the combination with triamcinolone lead to faster and longer-lasting improvements.

**Abstract:**

Osteoarthritis (OA) is a growing welfare problem for dogs and a challenge to manage for the clinician, and most therapeutic options aim to control pain. In a randomized, double-blinded, placebo-controlled, 2-way, 2-period crossover study, we aimed to evaluate the use of Blood Cell Secretome (BCS) administrated intra-articularly, with or without the combination with triamcinolone, in dogs with bilateral hip OA. BCS is an acellular orthobiologic containing anti-inflammatory and anabolic proteins produced from the patient’s own blood through extended coagulation in a defined environment. Fifteen dogs were initially assigned to a BCS group (BCSG, *n* = 5), a triamcinolone group (TG, *n* = 5), or a combination group (BCS+TG, *n* = 5). All had bilateral hip OA. After a 180-day follow-up, the crossover was performed with BCSG (*n* = 7) and BCS+TG (*n* = 7). BCSG received a single intra-articular administration of 3 mL of Blood Cell Secretome, and BCS+TG received BCS plus 0.5 mL of triamcinolone acetonide (40 mg/mL). The volume in BCSG was corrected to 3.5 mL with saline. In all patients, both hips were treated. For treatment follow-up, a copy of the Canine Brief Pain Inventory (divided into pain interference score—PIS and Pain Severity Score—PSS), Liverpool Osteoarthritis in Dogs (LOAD), and Canine Orthopedic Index (COI, divided into function, gait, stiffness, and quality of life) was completed on days 0, 8, 15, 30, 60, 90, 120, 150, and 180. Results were analyzed with the Mann–Whitney U test, effect size, and Kaplan–Meier estimators, followed by the log-rank test. *p* was set at <0.05. Patients of the sample had a mean age of 9.6 ± 2.9 years and a body weight of 29.2 ± 3.9 kg. Seven hips were classified as severe osteoarthritis, and eight were classified as moderate. No differences were found between groups at T0. Significant differences were observed in PSS scores at +8d, with BCS+TG exhibiting better results. PIS, PSS, LOAD, stiffness, and function scores were also lower in BCS+TG from +15 to +60d. The two groups showed similar improvements from +90 to +120d. Kaplan–Meier estimators showed that dogs in BCS+TG showed clinically-important differences for longer, despite a positive result in BCSG. The intra-articular administration of BCS alone was able to improve the overall condition of OA patients. Its combined use with triamcinolone acetonide lead to a faster and longer-lasting improvement in pain scores.

## 1. Introduction

Osteoarthritis (OA) is a disease commonly diagnosed in dogs, with studies pointing out a 20% prevalence in dogs over one year in North America and the United Kingdom [1]. The disease impacts the joint as a whole, along with its supporting tissues, but it is mostly associated with the loss of function of the articular cartilage [2]. OA affects companion and working dogs alike, having a significant toll on the animal’s overall quality of life and performance [3,4]. The existence of multiple therapeutic approaches to the management of OA illustrates the challenges of successfully treating the disease. The treatment can include intra-articular modalities [5,6,7,8,9], photobiomodulation [10], oral joint supplements [11], mesotherapy [12], biological products [13,14,15], or others.

Blood Cell Secretome (BCS) is an autologous biologic treatment. Its effect is obtained from a high concentration of anti-inflammatory mediators and growth factors. These include Interleukin 1 receptor antagonist (IL-1Ra), Transforming Growth Factor-β, and Insulin-like growth factor [16]. Additional factors, including lipid mediators and extracellular vesicles, may support the clinical effect of BCS [17]. OA has an important inflammatory component [18], and autologous BCS has been shown to reduce pain and increase weight-bearing in humans, with effects lasting up to 2 years [19], or at least one year of improvements in several clinical outcome measures [20,21]. This effect is obtained through a reduction in inflammatory mediators such as Interleukin-1b, nitric oxide, and reactive oxygen species [17,21]. Based on the observed clinical effects of BCS, its combined use with other products such as corticosteroids in a single IA administration may add increased benefits. The approach of combining corticosteroids with other products has been described with hyaluronan or PRP, aimed at providing a rapid onset of action, alongside a longer effect and a lower chance of side effects [6,22,23,24]. In a preliminary proof-of-concept study, we have reported that the combined use of BCS and the corticosteroid triamcinolone acetonide showed better effects than their isolated use [25].

Studies with a crossover design present some advantages compared to parallel group trials, as they increase study power relative to sample size by decreasing between-subject variability. In addition, they can facilitate simultaneous comparisons between and within groups [26]. We selected a crossover design for this study, which aimed to assess the intra-articular administration of BCS, alone or with triamcinolone, in dogs with OA. We hypothesized that the combined use would better alleviate joint pain while improving other OA-related clinical signs compared to BCS alone.

## 2. Materials and Methods

### 2.1. Studied Population

In this randomized, double-blinded, placebo-controlled, 2-way, 2-period crossover study, fifteen animals were enrolled. The sample was a convenience sample, constituted by dogs with bilateral hip OA. The diagnosis was made following history, physical, orthopedic, neurological, and radiographic examinations compatible with bilateral hip OA, and without signs or suspicion of any other disease [3]. In addition, dogs should be over two years old, have a body weight over 15 kg, and not have received any medication or nutritional supplement for over six weeks at the time of the initial enrolment [8]. The Orthopedic Foundation for Animals scheme was followed for hip grading [27].

**Figure 1:**
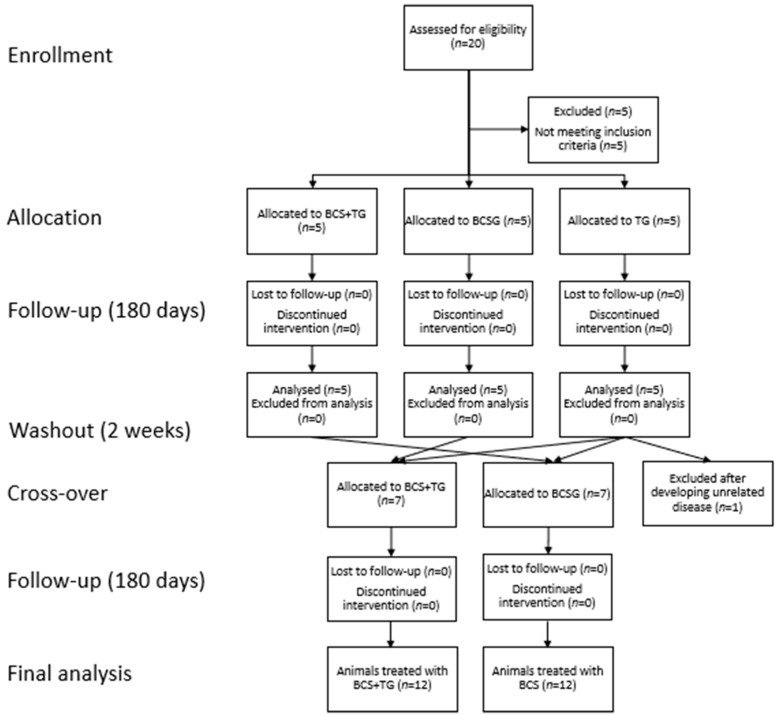
Full study flow diagram. Legend: BCSG—Blood Cell Secretome group; TG—Triamcinolone acetonide group; BCS+TG—Blood Cell Secretome + triamcinolone group.

After selection, for the first step of the crossover study, patients were randomly distributed between treatment groups. Five animals were assigned to a BCS group (BCSG), 5 to a triamcinolone acetonide group (TG), and 5 others to a BCS + triamcinolone group (BCS+TG). In all patients, both hips were treated. The results of this preliminary proof-of-concept study are reported elsewhere [25]. Following this first step and a 2-week washout period, the crossover was performed. Animals that were initially treated with BCS were now treated with BCS + triamcinolone group, and vice versa. Since TG showed significantly inferior results than BCSG and BCS+TG, but were in line with previous reports [8,9,28], they were equally and randomly divided between BCSG and BCS+TG and treated according to the assigned group. One animal in TG was not included in the second treatment stage after developing gastric dilation–volvulus and undergoing subsequent surgery. For the final analysis, data from 12 animals in BCSG and 12 animals in BCS+TG were considered. The full flow diagram is presented in Figure 1.

### 2.2. Treatment

The procedure for BCS preparation has been described before. Briefly, BCS was prepared with a commercially available kit (Orthogen^®^ Device, Orthogen AG, Düsseldorf, Germany), according to the manufacturer’s guidelines. Fifteen milliliters of whole blood were collected directly into the device from the jugular vein. Blood collection was performed early in the morning with the patient fasted. After blood collection, it was submitted to extended coagulation for 4.5 h at 37 °C (MF-6W incubator, HCP-Technology, Nortrup, Germany). After coagulation, a centrifugation stage was performed at 1500× *g* for 3 min (M-Universal, MPW, Warsaw, Poland). A vial containing the sterile filtered BCS was then collected. The BCS used in the second treatment was produced at the time of the first treatment and immediately frozen at −18 °C. It was kept frozen at the same temperature until the moment of use. For the second treatment in this crossover study, the sterile vial was removed from the freezer and thawed in the refrigerator overnight at 5 °C. All treatments were conducted early in the morning.

Dogs in BCSG were treated with a single intra-articular administration of 3 mL of BCS per hip joint. This volume was corrected to a final volume of 3.5 mL with saline, the same volume administered in BCS+TG. Patients in BCS+TG received a combined intra-articular administration of BCS (3 mL, prepared in the same way as previously described) and triamcinolone acetonide (20 mg in 0.5 mL, Triam Lichtenstein, Zentiva, Germany), amounting to a total volume of 3.5 mL. For all treatments, the syringe was covered to mask the administered treatment’s appearance. For the intra-articular administration, the dogs were placed under light sedation induced with a combination of medetomidine (0.01 mg/kg) and butorphanol (0.1 mg/kg), both given intravenously [14]. The intra-articular administration technique has been previously described [7]. With the dog in lateral recumbency, the limb of the joint being accessed is placed uppermost. Then, a window of 4 × 4 cm was clipped, with the greater trochanter in the center, and aseptically prepared. An assistant then placed the limb in a neutral position, parallel to the table. In this position, a 2,5″ 21-gauge needle was introduced just dorsal to the greater trochanter, perpendicular to the long axis of the limb, until the joint was reached. After confirmation that the needle was in the joint space through collection and removal of as much synovial fluid as possible, the treatment was administered.

### 2.3. Outcome Measures

Response to treatment was evaluated with validated clinical metrology instruments, the Canine Brief Pain Inventory (CBPI, divided into pain severity score—PSS and pain interference score—PIS), the Canine Orthopedic Index (COI, divided into stiffness, gait, function, and quality of life—QOL), and the Liverpool Osteoarthritis in Dogs (LOAD). All have been previously validated in their Portuguese versions [29,30,31]. A digital copy of these clinical metrology instruments was completed by the dog’s handlers on treatment days 8, 15, 30, 60, 90, 120, 150, and also 180 days post-treatment. The same dog handlers, blinded to treatment, completed all questionnaires for each dog throughout the entire study. They were not shown their previous answers before completing a new copy of the questionnaires.

### 2.4. Statistical Analysis

Normality was assessed with a Shapiro–Wilk test. In each evaluation moment, groups were compared using a Mann–Whitney U test. The effect size was also determined, and a value of <0.3 was considered a small effect, a value between 0.3 and 0.5 a medium effect, and values >0.5 a strong effect [32]. Time-to-event curves were created with the Kaplan–Meier test. Event probability was compared with the log-rank test. Based on what constitutes a clinically important difference for each clinical metrology instrument, specific measures of success were defined. With the CBPI, it has been set as a reduction of ≥1 in PSS and ≥2 in PIS [33]. For the LOAD, a measure of success has been set as a reduction of ≥4 [34]. For the COI, the success level has only been determined for the overall score, as a reduction of ≥3.5 [34]. For these scores, the time for the improvement to reduce below this level was evaluated. For the analysis of the dimensions of the COI (stiffness, gait, function, and QOL), we considered the event as the moment where an improved score, compared to baseline values, was no longer observed. We selected this event since it was the moment when medical assistance was sought for the animal, meaning it has some level of clinical significance [35,36]. Patients with improved scores above the described levels at the final evaluation moment were censored. Data were analyzed with IBM SPSS Statistics version 20. A *p* of <0.05 was set.

## 3. Results

The sample included 15 active police working dogs, both males (*n* = 8) and females (*n* = 7), with a mean age of 9.6 ± 2.9 years and bodyweight of 29.2 ± 3.9 kg. The breeds represented are among the ones most commonly used as police working dogs and included 11 German Shepherd Dogs, two Labrador Retrievers, one Belgian Malinois Shepherd Dog, and one Dutch Shepherd Dog. Concerning hip grading, 8 hips were classified as moderate OA and 7 as severe. The classification did not change throughout the study period. One animal of TG was removed from the study during the washout period after developing gastric dilation–volvulus. The remaining dogs were followed up to the two last evaluation moments. Enough BCS was obtained to treat both hips at both treatment moments (at least 6 mL of BCS). No additional treatment or medications were administered, and no side effects were observed.

**Figure 2:**
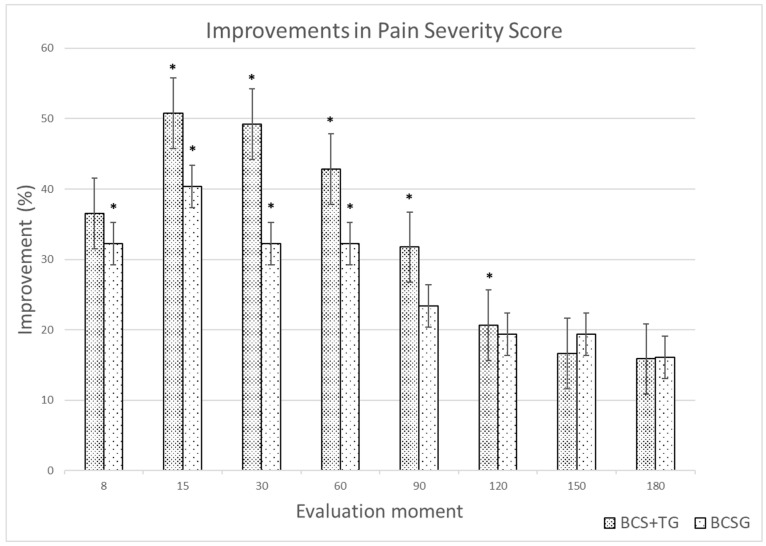
Improvements (%) in Pain Severity Score for the Blood Cell Secretome + triamcinolone group (BCS+TG) and the Blood Cell Secretome group (BCSG), compared to baseline values. * indicates a clinically significant improvement (reduction ≥ 1).

**Figure 3:**
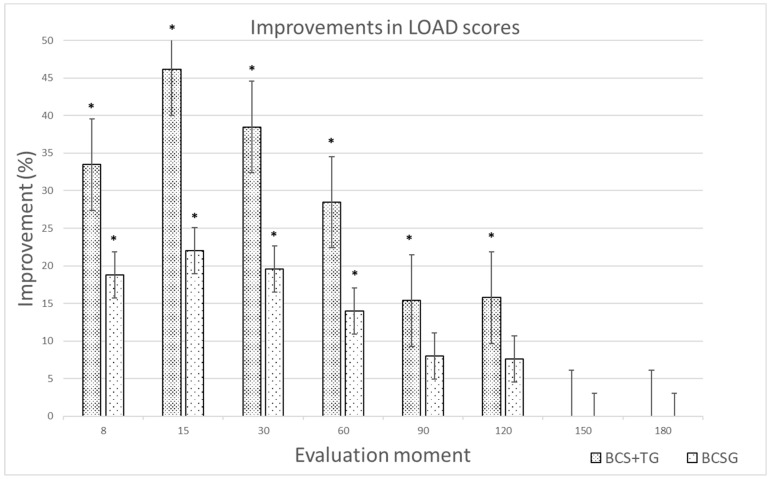
Improvements (%) in Liverpool Osteoarthritis in Dogs (LOAD) scores for the Blood Cell Secretome + triamcinolone group (BCS+TG) and the Blood Cell Secretome group (BCSG), compared to baseline values. * indicates a clinically significant improvement (reduction ≥ 4).

The results of the CBPI, the LOAD, and the COI up to the +60d evaluation are presented in Table 1. The results from the +90d to the +180d evaluation moments are presented in Table 2. Significant differences were observed between groups in PSS scores from day 8 after treatment and in both scores of the CBPI from the +15d evaluation up to the +60d evaluation, with BCS+TG showing lower scores. The same was observed with the LOAD. With the stiffness and function scores, a difference was observed from the +15d to +60d period. At the +90d to +120d period, the two groups experienced a similar level of improvement. At the +120d evaluation, BCS+TG had better LOAD and function scores and at the +150d had better PIS scores. Clinically significant improvements were observed in the two groups compared to results at T0. The improvements in PSS and LOAD for each group are presented in Figure 2 and Figure 3, respectively.

**Figure 4:**
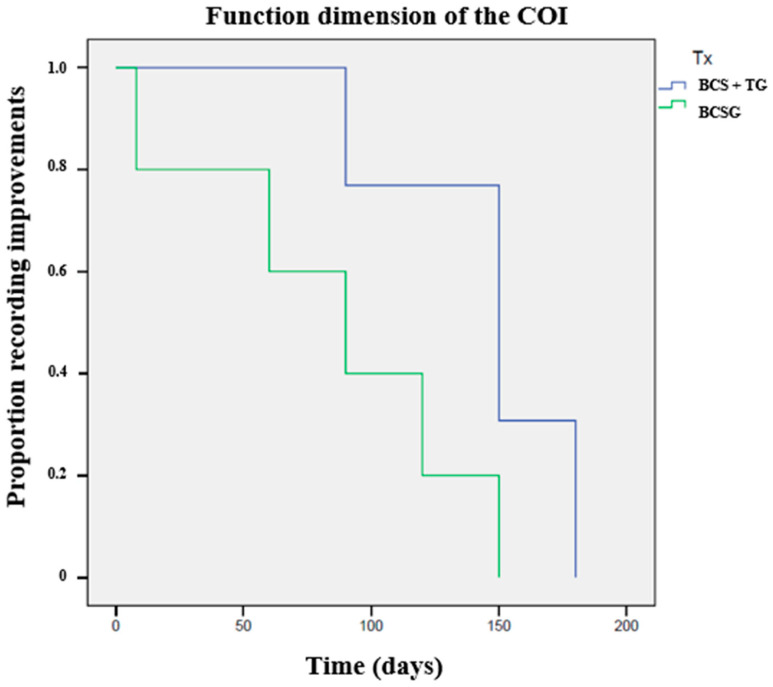
Kaplan–Meier curves demonstrating a significant difference between the Blood Cell Secretome + triamcinolone group (BCS+TG) and the Blood Cell Secretome group (BCSG), in time (days) for the improvement in Function score to reduce below baseline values (*p* < 0.01, compared with the logrank test).

Results of the Kaplan–Meier time-to-event estimators are presented in Table 2. The evolution of COI’s Function dimension is presented in Figure 4. Dogs in BCS+TG took longer to record the considered events.

## 4. Discussion

Managing OA remains a challenge for clinicians, and there is an ongoing need for therapeutic options that address the complexity of the disease [37,38]. The results of this study reinforce the concept that the intra-articular administration of BCS improves the clinical signs of the disease, as measured with several clinical metrology instruments, and that the combination of BCS with triamcinolone produces better clinical results than BCS alone.

Previous reports on different autologous blood products sharing a similar action mode to BCS described a positive effect in OA patients, reducing pain, improving lameness scores, and increasing weight bearing [39,40,41]. These results proved to be superior to placebo or hyaluronan in several clinical outcome measures [19,21]. The duration of these improvements varies between reports but have been reported as lasting up to 12 weeks [40,42]. The results of the present report confirm the findings of the original preliminary report [25], showing that BCS has a superior effect compared to triamcinolone and improves the scores of several clinical metrology instruments, particularly when combined with triamcinolone. These improvements can be observed in both BCSG and BCS+TG from the first follow-up and last for a long period. A difference between the two groups can be observed from the first evaluation point, specifically regarding pain scores. This effect is likely due to the effect of triamcinolone, which introduces a great anti-inflammatory effect in the joint environment. BCS+TG also recorded better results with the LOAD, function, and stiffness, with a significant result being observed in the effect size calculation. This difference between groups was not evident at the +90d evaluation moment, and for most scores at the +120d, despite both groups recording improvements compared to baseline values at a level considered as significant improvement for these clinical metrology instruments [32,33]. Interestingly, a significant difference was observed for PIS at the +150d evaluation point. It is possible that the addition of triamcinolone, and its effects in reducing inflammation, may provide a better action ground for BCS. This appears to be confirmed by the results of the Kaplan–Meier analysis, where many scores in BCS+TG were significantly better up to the +120d to +150d follow-up period, despite BCSG recording a mean time to event around the +120d evaluation moment. This overall longevity in improvements following treatment is relevant, specifically as almost half of the animals in the sample had severe OA, and since with the crossover study a whole year was covered, with all the variations in conditions it implies, particularly the effect of cold weather on OA clinical signs.

There are several guidelines for the management of OA in humans, which provide recommendations for the use of intra-articular corticosteroids [43,44,45,46,47]; there is still a concern that they may have a deleterious effect on joint tissues [48]. Unpublished data from the manufacturer of the device used to produce BCS shows that BCS has a protective effect over the possible deleterious effect of corticosteroids in a culture of human chondrocytes. It would be important to have similar information published on a possible protective effect of BCS in canine chondrocytes to evaluate if the positive clinical effect we observed is matched at the cellular level.

There are some side effects documented after intra-articular administrations, including local pain and local inflammation, although these are usually self-limiting [34]. While some patients in this study showed complaints for a couple of days following the intra-articular administration in all groups, these resolved spontaneously. The study presents some limitations. We used a convenience sample, so future studies should include a sample size calculation. They should also include an objective evaluation, such as Force Plate Gait Analysis or Stance Analysis. The molecular characterization of BCS to identify components that either enhance or reduce effect is also of interest. For future studies, the evaluation of the BCS-effect on synoviocytes, chondrocytes, and synovial fluid inflammatory markers should be considered.

## 5. Conclusions

Our results showed that the intra-articular administration of BCS was able to improve the overall condition of OA patients, as measured with several validated clinical metrology instruments. Although BCS alone was able to produce this effect, its combined use with triamcinolone acetonide lead to a greater improvement in pain scores, and the observed results lasted longer.

## Figures and Tables

**Table 1:** Evolution of Clinical Metrology instruments (median score, interquartile range, percentual variation, and effect size), by group, up to the +60 days evaluation moment. CBPI—Canine Brief Pain Inventory; COI—Canine Orthopedic Index; LOAD—Liverpool Osteoarthritis in Dogs; PIS—Pain Interference Score; PSS—Pain Severity Score; QOL—Quality of Life. * indicates significance when comparing groups at each follow-up moment.

Clinical Metrology Instrument		Group	T0		*p*	+8d			*p*	ES	+15d			*p*	ES	+30d			*p*	ES	+60d			p	ES
			Med	IQR		Med	IQR	%			Med	IQR	%			Med	IQR	%			Med	IQR	%		
CBPI	PSS (0–10)	BCS+TG	6.3	1.0	0.93	4.0	2.0	36.5	0.03 *	0.3	3.1	2.2	50.8	0.02 *	0.4	3.2	2.0	49.2	0.02 *	0.3	3.6	1.3	42.9	0.04 *	0.3
		BCS	6.2	1.3		4.2	1.6	32.3			3.7	0.6	40.3			4.2	0.8	32.3			4.2	2.0	32.3		
	PIS (0–10)	BCS+TG	6.0	1.8	0.17	4.8	2.2	20.0	0.06	0.2	2.9	2.5	51.7	0.01 *	0.4	3.1	2.0	48.3	0.02 *	0.3	3.6	1.2	40.0	0.44	0.2
		BCS	5.9	1.6		4.8	1.1	18.6			3.4	1.1	42.4			3.6	0.8	39.0			3.8	1.3	35.6		
LOAD (0–52)		BCS+TG	26.0	5.0	0.55	17.3	3.0	33.5	0.04 *	0.3	14.0	1.0	46.2	0.01 *	0.4	16.0	3.0	38.5	0.04 *	0.2	18.6	2.0	28.5	0.04 *	0.3
		BCS	25.0	7.3		20.3	4.8	18.8			19.5	5.0	22.0			20.1	5.5	19.6			21.5	4.5	14.0		
COI	Stiffness (0–16)	BCS+TG	8.0	1.0	0.56	5.9	3.0	26.3	0.63	0.1	4.2	3.0	47.5	0.03 *	0.3	4.2	1.0	47.5	0.03 *	0.3	5.2	2.0	35.0	0.03 *	0.3
		BCS	7.5	2.8		6.1	2.0	18.7			5.2	3.8	30.7			5.2	4.0	30.7			5.8	2.8	22.7		
	Function (0–16)	BCS+TG	8.0	2.0	0.87	6.0	2.0	25.0	0.6	0.1	4.0	3.0	50.0	0.02 *	0.4	4.0	1.0	50.0	0.02 *	0.4	6.0	1.0	25.0	0.03 *	0.2
		BCS	8.0	4.5		6.0	1.0	25.0			6.0	1.8	25.0			6.0	1.8	25.0			6.8	2.3	15.0		
	Gait (0–20)	BCS+TG	12.0	3.0	0.75	9.3	4.0	22.5	0.27	0.2	10.0	5.0	16.7	0.34	0.2	10.0	4.0	16.7	0.34	0.2	10.0	1.0	16.7	0.80	0.1
		BCS	11.5	3.5		10.0	1.8	13.0			10.5	1.0	8.7			10.5	1.8	8.7			11.0	4.0	4.3		
	QOL (0–12)	BCS+TG	7.0	1.0	0.95	5.7	3.0	18.6	0.88	0.0	6.0	4.0	14.3	0.93	0.0	6.0	1.0	14.3	0.93	0.0	7.0	3.0	0.0	0.69	0.1
		BCS	6.5	1.0		6.0	1.8	7.7			6.0	1.8	7.7			6.0	2.0	7.7			6.0	1.0	7.7		
	Overall (0–64)	BCS+TG	35.0	4.0	0.98	26.9	10.0	23.1	0.56	0.1	24.2	14.0	30.9	0.04 *	0.3	24.2	6.0	30.9	0.04 *	0.3	28.2	1.0	19.4	0.93	0.0
		BCS	33.5	10.8		28.1	6.8	16.1			27.7	6.5	17.3			27.7	5.5	17.3			29.6	11.8	11.6		
Clinical Metrology Instrument		Group	+90d					*p*	ES		+120d			*p*	ES	+150d			*p*	ES	+180d			*p*	ES
			Med			IQR	%				Med	IQR	%			Med	IQR	%			Med	IQR	%		
CBPI	PSS (0–10)	BCS+TG	4.3			2.8	31.7	0.93	0.0		5.0	2.0	20.6	0.95	0.0	5.3	1.4	16.7	0.51	0.1	5.3	1.8	15.9	0.30	0.2
		BCS	4.8			1.3	23.4				5.0	0.9	19.4			5.0	1.9	19.4			5.2	1.6	16.1		
	PIS (0–10)	BCS+TG	4.0			2.5	33.3	0.62	0.1		5.0	1.0	16.7	0.66	0.1	4.8	1.8	20.0	0.04 *	0.3	6.0	1.3	0.0	0.59	0.2
		BCS	3.9			0.9	33.9				5.1	0.9	13.6			5.4	0.6	8.5			5.8	0.9	1.7		
LOAD (0–52)		BCS+TG	22.0			6.0	15.4	0.66	0.1		21.9	4.0	15.8	0.04 *	0.2	26.0	6.0	0.0	0.55	0.1	26.0	7.0	0.0	0.58	0.1
		BCS	23.0			6.0	8.0				23.1	4.5	7.6			25.0	3.8	0.0			25.0	3.8	0.0		
COI	Stiffness (0–16)	BCS+TG	6.2			5.0	22.5	0.68	0.1		7.0	3.0	12.5	0.90	0.0	8.0	2.0	0.0	0.57	0.1	8.0	1.0	0.0	0.97	0.0
		BCS	6.3			1.0	16.0				7.5	2.5	0.0			7.0	3.0	6.7			8.0	1.0	-6.7		
	Function (0–16)	BCS+TG	6.0			3.0	25.0	0.88	0.2		7.0	2.0	12.5	0.03 *	0.3	8.0	5.0	0.0	0.98	0.0	8.0	6.0	0.0	0.64	0.1
		BCS	6.8			0.8	15.0				7.7	1.0	3.8			8.0	1.8	0.0			8.0	0.8	0.0		
	Gait (0–20)	BCS+TG	10.0			5.0	16.7	0.75	0.1		12.0	3.0	0.0	0.41	0.2	12.0	3.0	0.0	0.45	0.2	13.0	2.0	-8.3	0.16	0.3
		BCS	10.0			0.8	13.0				10.0	1.0	13.0			10.0	3.5	13.0			11.0	3.3	4.3		
	QOL (0–12)	BCS+TG	6.0			3.0	14.3	0.87	0.0		7.0	0.0	0.0	0.07	0.2	7.0	2.0	0.0	0.46	0.2	7.0	1.0	0.0	0.24	0.2
		BCS	6.0			0.0	7.7				6.0	0.0	7.7			7.0	1.0	−7.7			6.5	1.0	0.0		
	Overall (0–64)	BCS+TG	28.2			16.0	19.4	0.48	0.1		33.0	8.0	5.7	0.25	0.2	35.0	11.0	0.0	0.53	0.1	36.0	9.0	−2.9	0.23	0.3
		BCS	29.1			2.8	13.1				31.2	3.5	6.9			32.0	5.0	4.5			33.5	5.5	0.0		

**Table 2:** Time-to-event (in days) for the clinical metrology instruments considered in the Blood Cell Secretome + triamcinolone group (BCS+TG) and Blood Cell Secretome group (BCSG), calculated with Kaplan–Meier estimators and compared with the logrank test. Events considered were a score change below the improvement level set as clinically significant for each score: ≥1 in PSS, ≥2 in PIS [33], ≥4 in LOAD [34], ≥3.5 in COI [34], or when an improved score, compared to baseline values, was no longer observed for stiffness, function gait, and QOL. Legend: CBPI—Canine Brief Pain Inventory; CI—Confidence interval; COI—Canine Orthopedic Index; LOAD—Liverpool Osteoarthritis in Dogs; PIS—Pain Interference Score; PSS—Pain Severity Score; QOL—Quality of Life. * indicates significance.

Clinical Metrology Instrument		Log-Rank Test	BCS+TG				BCSG			
			Mean	SD	95% CI		Mean	SD	95% CI	
CBPI	PSS	0.02 *	154.6	6.4	142.1	167.2	115.3	16.9	86.7	153.3
	PIS	<0.01 *	166.2	4.4	157.6	174.7	112.0	4.9	92.4	111.6
LOAD		0.42	121.6	19.9	82.6	160.5	118.9	17.3	84.9	152.9
COI	Stiffness	0.32	120	10.4	99.6	140.4	91.8	15.2	62.0	121.6
	Function	<0.01 *	145.4	9.5	126.7	164.0	90.1	16.3	53.6	117.6
	Gait	<0.01 *	172.3	6.7	145.9	180.0	123	10.2	112.0	151.9
	QOL	<0.01 *	131.5	4.2	123.3	139.8	95.2	10.9	68.5	111.5
	Overall	0.02 *	175.4	3.2	169.0	181.7	123.5	12.9	106.8	157.2

## Data Availability

All data generated or analyzed during this study are included in this published article.
